# Anti-Tumor Drug-Loaded Oxygen Nanobubbles for the Degradation of HIF-1α and the Upregulation of Reactive Oxygen Species in Tumor Cells

**DOI:** 10.3390/cancers11101464

**Published:** 2019-09-29

**Authors:** Muhammad Saad Khan, Jangsun Hwang, Kyungwoo Lee, Yonghyun Choi, Youngmin Seo, Hojeong Jeon, Jong Wook Hong, Jonghoon Choi

**Affiliations:** 1 School of Integrative Engineering, Chung-Ang University, Seoul 06974, Korea; saad.engr@gmail.com (M.S.K.); isnickawesome@gmail.com (J.H.); orztapa@gmail.com (K.L.); dydgus5057@gmail.com (Y.C.); 2 Center for Biomaterials, Korea Institute of Science and Technology, Seoul 02792, Korea; drslow@kist.re.kr (Y.S.); jeonhj@kist.re.kr (H.J.); 3 Department of Bionano Technology, Hanyang University, Seoul 426-791, Korea; jwh@hanyang.ac.kr; 4 Department of Bionano Engineering, Hanyang University, Ansan 426-791, Korea

**Keywords:** doxorubicin, drug delivery, oxygen nanobubbles, hypoxia, hypoxia-inducible factor 1 alpha

## Abstract

Hypoxia is a key concern during the treatment of tumors, and hypoxia-inducible factor 1 alpha (HIF-1α) has been associated with increased tumor resistance to therapeutic modalities. In this study, doxorubicin-loaded oxygen nanobubbles (Dox/ONBs) were synthesized, and the effectiveness of drug delivery to MDA-MB-231 breast cancer and HeLa cells was evaluated. Dox/ONBs were characterized using optical and fluorescence microscopy, and size measurements were performed through nanoparticle tracking analysis (NTA). The working mechanism of Dox was evaluated using reactive oxygen species (ROS) assays, and cellular penetration was assessed with confocal microscopy. Hypoxic conditions were established to assess the effect of Dox/ONBs under hypoxic conditions compared with normoxic conditions. Our results indicate that Dox/ONBs are effective for drug delivery, enhancing oxygen levels, and ROS generation in tumor-derived cell lines.

## 1. Background

Hypoxia, or lack of oxygen, is a common characteristic of solid tumors, and it has been correlated with poor treatment outcomes [1,2,3,4,5]. Tumors grow rapidly, and there is often an imbalance in the supply of and demand for oxygen and nutrients, creating hypoxic conditions and decreasing oxygen saturation levels into the range of 1% to 5%. [1,6,7]. Tumors under hypoxic conditions exhibit higher resistance to chemotherapy, radiotherapy, and photodynamic therapy [6,8,9]. Hypoxic conditions stabilize hypoxia-inducible factor-1 alpha (HIF-1α) protein, which has been reported to be a cause of tumor resistance to therapeutic mechanisms [7,10,11,12,13,14,15,16,17,18]. Within tumor cells, HIF-1α has been associated with a shift toward anaerobic metabolism and the expression of numerous genes involved in angiogenesis, apoptosis, pH regulation, and cellular differentiation. Therefore, many studies are focused on downregulating or suppressing HIF-1α to increase treatment efficacy [10,16,19,20,21,22,23].

Microbubbles and nanobubbles are composed of a stabilizing monolayered shell and a gas core, and have a long history of use as contrast agents in ultrasound [24,25,26,27,28,29,30,31,32]. Over the past two decades, more attention has been paid to the use of microbubbles for therapeutic purposes because of their biocompatibility, smaller size, ability to deliver drugs and gases, higher surface contact area, and variety of compositions [33,34,35,36,37,38,39]. Various researchers have used microbubbles and nanobubbles to deliver oxygen gas to overcome hypoxia and hypoxemia [28,40,41,42,43,44,45,46]. Nanosized particles with modified surfaces that encapsulate drugs and other therapeutic agents have been applied to therapeutic purposes based on their size-dependent physical properties [47,48,49]. Nanobubbles and microbubbles have similar gas/core compositions, but nanobubbles provide an additional advantage of a smaller size, which makes them more favorable for therapeutic applications [26,50,51,52,53,54]. Nanobubbles can be loaded with drugs by either encapsulating a hydrophobic drug inside the core or by associating the drug with the shell in a covalent or non-covalent manner [26,55,56,57]. Nanobubbles can be actively targeted by incorporating targeting ligands such as antibodies on their surface. They can also be used passively targeting by exploiting the mechanism of enhanced permeability and retention (EPR), because they are small enough to pass through endothelial gaps created in the tumor vasculature [2,26,51,58,59]. Nanobubbles are different from liposomes, because nanobubbles have a gas core within a monolayer shell [38,60,61]. Phospholipids have been investigated as encapsulating shells for microbubbles and nanobubbles [25], because they are easy to synthesize and remain stable at the nanoscale while having a variety of functionalization possibilities [26,62]. Lipid-shelled bubbles are biodegradable after they are taken up by cells through endocytosis; therefore, ultrasound exposure is not required for their destruction. Incorporating polyethylene glycol (PEG) in the bubble shells increases their half-life, circulation time, and biocompatibility [63,64,65,66,67], and a longer circulation time enhances the passive targeting and retention ability of the drug, improving its biodistribution [68].

Doxorubicin (Dox) belongs to the anthracycline class of chemotherapeutic drugs, and is commonly used for the treatment of various cancers, including breast cancer and prostate cancer [69,70]. The chemotherapeutic mechanism of Dox is based on the intercalation with DNA, inhibition of the critical DNA replicating enzyme topoisomerase II, and production of reactive oxygen species (ROS) [69,70,71,72]. Several side effects of free Dox have been reported, and are related to cardiac toxicity and cardiomyopathy [70,73,74]. Therefore, to reduce the side effects of Dox and improve the efficiency and effectiveness of Dox delivery, liposome-encapsulated Dox has been developed for commercial use [71,75]. Dox is an amphiphilic compound; therefore, it can be embedded in phospholipid shells through electrostatic and hydrophobic interactions [76]. Liposomes are composed of lipid bilayers with an aqueous core, and they are used for drug delivery applications because of their echogenicity, high drug-loading capacity, and targeting ability, as well as their ability to reduce the side effects of incorporated drugs such as Dox. Dox release from a liposomal formulation can be measured directly because of the intrinsic Dox fluorescence, because the fluorescence signal of Dox decreases when encapsulated in liposomes and increases following the release of Dox [71]. Liposomal Dox exhibits altered pharmacokinetics and biodistribution compared to free Dox, increasing passive targeting of the EPR effect and reducing its cardiotoxicity. Liposome conjugation slows the drug release rate and improves the bioavailability of Dox for a more extended period [71,77,78,79].

For Dox delivery applications, research groups have demonstrated the use of ultrasound for the destruction of Dox-conjugated microbubbles [53,75,76]. Dox delivery into tumor cells using ultrasound while co-administering free Dox and microbubbles has also been demonstrated by Escoffre et al. [69]. In both of these mechanisms, ultrasound plays a crucial role in destroying microbubbles and enhancing the permeability of the cells to enable Dox delivery.

Another mechanism of the chemotherapeutic action of Dox relies on ROS generation, which requires the presence of sufficient oxygen at the tumor site, so increasing oxygen levels will improve the efficacy of Dox. Moreover, an increase in oxygen saturation might enhance the efficacy of Dox, because hypoxic conditions and HIF-1α protein activity have been shown to cause Dox in tumors [19,20,21,22,72,80,81,82].

Our group has demonstrated that oxygen nanobubbles (ONBs) reverse hypoxia and suppress HIF-1α activity in tumor cells (ONBs) [38]. We also reported the surface composition, preparation methods, and stability of oxygen nanobubbles using phospholipid shells in a previous study [32]. In this study, we synthesized Dox-conjugated ONBs (Dox/ONBs) to increase Dox effectiveness in hypoxic conditions by suppressing HIF-1α activity and increasing ROS generation. We used phospholipids for the shell composition to encapsulate Dox, owing to the ease of synthesis, biocompatibility, and biodegradability. Synthesizing nanosized bubbles was aimed at achieving cellular penetration without using ultrasound. We presumed that conjugating drugs to nanobubbles could simplify the drug delivery process, because nanobubbles can penetrate the tumor. We hypothesized that this method might suppress hypoxia-induced tumor resistance, increase Dox-induced ROS, and reduce the amount of drug needed to achieve an effective clinical outcome, thereby reducing the side effects of free Dox.

## 2. Materials and Methods

### 2.1. Materials

All lipids, namely, 1,2-distearoyl-sn-glycero-3-phosphocholine (DSPC), 1,2-distearoyl-sn-glycero-3-phosphoethanolamine-N-[amino(polyethylene glycol)-2000] (DSPE-PEG-2000-Amine), and 1,2-distearoyl-sn-glycero-3-phosphoethanolamine-N-[biotinyl(polyethylene glycol)-2000] (DSPE-PEG-2000-Biotin) were purchased from Avanti Polar Lipids (Alabaster, AL, USA). An OxiSelect intracellular ROS assay kit for the fluorometric detection of ROS was purchased from Cell Biolabs Inc. (San Diego, CA, USA). A 99.995% pure oxygen cylinder (Daesung gas, Siheung-si, Gyeonggi-do, South, Korea) was used for oxygen supply during the synthesis of Dox/ONBs. Doxorubicin-HCL, chloroform, cell culture media, and other reagents were purchased from Sigma-Aldrich (St. Louis, MO, USA). DoxovesTM (Liposomal Doxorubicin-HCl) was purchased from FormuMax Scientific (Sunnyvale, CA, USA).

### 2.2. ONB Characterization

The detailed synthesis of ONBs, their characterization, and measuring their ability to deliver oxygen to hypoxic regions and downregulate the HIF-1α protein have been explained in our previous work [38]. In the present study, we altered the synthesis of ONBs by incorporating Dox, as described below.

### 2.3. Cell Penetration of Dox/ONBs

First, different combinations of DSPC, DSPE-PEG-2000-Amine, and DSPE-PEG-2000-Biotin were used to synthesize ONBs, and Dox was co-administered by mixing it with these different ONBs to determine the optimal combinations of lipid constituents for effective penetration into cells. DSPC, DSPE-PEG-Amine, and DSPE-PEG-Biotin were each used to synthesize ONBs at ratios of 50:50:0, 80:20:0, and 85:8:7. The same amount of Dox was mixed with each bubble composite and introduced into the media of MDA-MB-231 cells cultured in 96-well plates. After 6 h of incubation, cells were washed with Dulbecco’s phosphate-buffered saline (DPBS), and Dox fluorescence was measured for excitation/emission at 480/560 nm with a microplate reader.

Cell penetration of ONBs containing DSPE-PEG-2000-Biotin (85:8:7 composite) was tested similarly by preparing fluorescein (FITC)-conjugated ONBs. Since DSPE-PEG-2000-Biotin is a component of this ONB shell, avidin–biotin interaction was utilized for fluorescent ONBs and 100 µL FITC-avidin was added to ONBs and centrifuged at 300× *g* for 10 minutes to allow avidin-biotin interaction. Cells were washed after a 2-h incubation at 37 °C with free FITC and FITC/ONB. FITC fluorescence was measured at 480/520 nm.

### 2.4. Dox/ONB Preparation

Dox-conjugated ONBs were prepared by first dissolving 25.2 mg of DSPC, 8.4 mg of DSPE–PEG–Amine, and 7.95 mg of DSPE–PEG–Biotin (molar ratio 85:8:7) with 5 mg of Dox in chloroform in a flask and drying in a hot-air oven at 70 °C. The dried layer was rehydrated by adding 10 mL of DPBS followed by sonication in a bath at a temperature above 60 °C. Then, the suspension was sonicated again in the presence of oxygen supply (99.9% oxygen cylinder) using a tip sonicator at 190 W. The suspension was centrifuged at 300× *g*, and the visible bubbles at the top were discarded. A 1-µm syringe filter (MerckMillipore, Kenilworth, NJ, USA) was used to discard microbubbles. Then, the suspension was dialyzed against deionized water using a 1400-Da dialysis tube against deionized water for two days under constant stirring to remove unbound Dox. After dialysis, the suspension was recollected into a conical tube and reoxygenated using an oxygen supply.

### 2.5. Characterization of Dox/ONB

Optical and fluorescence microscopy was used to visualize the core–shell composition of the microsized bubbles. The Dox content was calculated by first making a Dox standard curve using Dox fluorescence at 480/560 nm, and then calculating the fluorescence of Dox/ONB against the standard curve. The size determination of nanosized bubbles was performed with a nanoparticle tracking (NTA) (Nanosight NS 300, Malvern, PA, USA) system. The size changes of ONBs due to variation in pH was evaluated through buffer solutions of pH 2, 4.5, 6.5, and 7.4. ONBs were diluted 1:1000 (v/v) in these buffer solutions to measure the number of particles and size using NTA. Zeta potential was measured using dynamic light scattering (DLS) (Malvern, PA, USA). Drug release from Dox/ONBs was tested by injecting the drug into dialysis tubing and dialyzing against DPBS at 37 °C. At each time point, 1 mL of DPBS was removed and replaced with fresh DPBS, and fluorescence was measured to quantify the amount of drug released using a Dox standard curve.

Cell penetration of Dox was then assessed by confocal laser scanning microscopy (LSM 710, Carl Zeiss, Oberkochen, Germany). MDA-MB-231 and HeLa cells were seeded onto an 8-well glass slide. Free Dox and Dox/ONBs were introduced, and samples were fixed with 99% ethanol after 1 min, 30 min, 2 h and 6 h. Samples were stained with 4′,6-Diamidino-2-phenylindole dihydrochloride (DAPI) to visualize the nucleus.

### 2.6. ROS Assay to Determine the Dox Mechanism

An ROS assay kit was used to determine the Dox mechanism in MDA-MB-231 and HeLa cells. Cells were seeded in a 96-well plate, and after 24 h, 0.1× 2′,7′-Dichlorofluorescin Diacetate (DCFH-DA) was applied for 1 h. Cells were washed with DPBS and treated with ONB, Dox, Dox (2×), Doxoves, Dox + ONB and Dox/ONBs for 6 h. While Dox (2×) has twice amount of drug as compared to Dox. Fluorescence was measured for excitation/emission at 480/530 nm. H_2_O_2_ (Cell Biolabs Inc., San Diego, CA, USA) was used as a positive control standard for ROS generation.

### 2.7. Dox Efficacy Under Normal and Hypoxic Conditions (MDA-MB-231 Cells and HeLa Cells)

To assess Dox efficacy in normal conditions, cells were seeded in a 24-well plate and kept in a standard incubator at 37 °C for 24 h. For normal conditions, Dox concentration was used at 1.3 µg/mL for free Dox, Doxoves, Dox + ONB mixture, and Dox/ONB. Hypoxic conditions were created as described in our previous work. Briefly, cells were seeded in 6-well or 24-well plates, and the plate was kept in a sealable chamber. Argon gas was purged through the chamber to remove the air inside. We reported the successful creation of a hypoxic environment and the reversal of hypoxia using this protocol [33]. In the current set of experiments, tumor cells were kept under hypoxic conditions for 6–8 h and then treated with free Dox, Doxoves, ONB, and Dox/ONB to evaluate the effect of hypoxic conditions on cell viability. For hypoxic conditions, Dox concentration was used at 13 μg/mL for free Dox, Doxoves, Dox + ONB, and Dox/ONB. The result of bioconjugation was assessed by comparing the effects of Dox/ONB with those of similar concentrations of Dox and ONB administered together, which is indicated as Dox + ONB in the Results section.

Cell viability was measured by resuspending the cells using trypsin and staining them with 4% trypan blue. An automated cell counter (Juli-Br, NanoEntek, Korea) was used to measure cell viability (%).

### 2.8. Statistical and Image Analyses

Dox uptake was measured by taking confocal microscopy images of MDA and HeLa cells. The fluorescence intensity was quantified using ImageJ software. Corrected total cell fluorescence (CTCF) was measured using the following formula:

CTCF = Integrated density − (Area of selected cells × Mean fluorescence of background reading)

The same area of cells was selected to quantify fluorescence. At least 10 measurements were taken, and CTCF was calculated using ImageJ software (National Institute of Health, Bethesda, MD, USA). Graphs were made using the GraphPad Prism (Graphpad, La Jolla, CA, USA). Statistical analysis was performed using one-way ANOVA, followed by post hoc analysis with Tukey’s multiple comparisons test using GraphPad Prism. Asterisks (*, **, ***, and ****) denote *p*-values of < 0.05, 0.01, 0.001 and 0.0001, respectively.

## 3. Results

**Figure 1:**
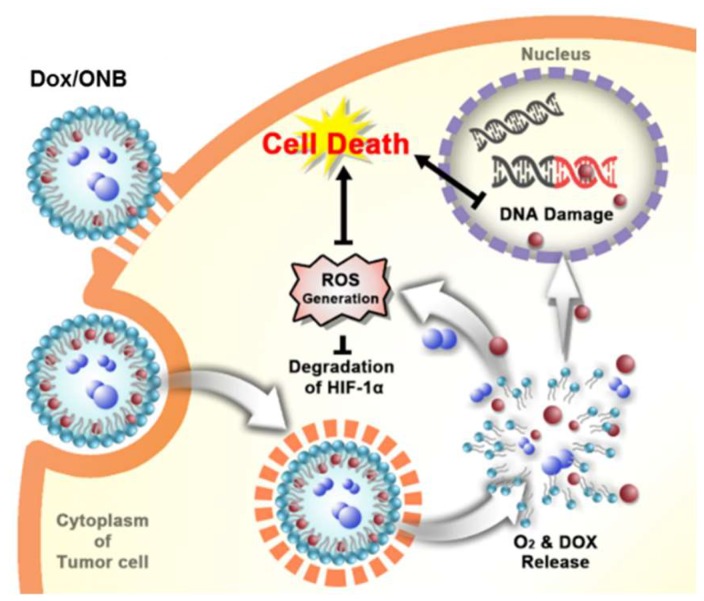
Schematic of doxorubicin-loaded oxygen nanobubble (Dox/ONB) and the working mechanism of Dox/ONBs inside a tumor cell.

The scheme for the mechanism of Dox/ONBs inside a tumor cell is depicted in Figure 1. When injected into a tumor site, Dox/ONBs are taken up by cells through endocytosis, and oxygen and Dox are released after metabolism by the cell. An increase in intracellular oxygen leads to the degradation of the HIF-1α protein, which in turn, reduces tumor resistance against the drug. More ROS are generated in the presence of oxygen, and the DNA damage resulting from the action of Dox results in cell death. The encapsulation of Dox in Dox/ONBs reduces the side effects of Dox, and the presence of oxygen increases its efficacy, thereby requiring a lower amount of drug to achieve its intracellular effects. Dox from Dox/ONB is primarily released via a diffusion mechanism. When the gas diffuses out of nanobubbles, it shrinks, increasing the Laplace pressure and collapsing the nanobubble. Additionally, the collapsed nanobubbles are taken up by the cells, and Dox is released as a result of metabolism.

**Figure 2:**
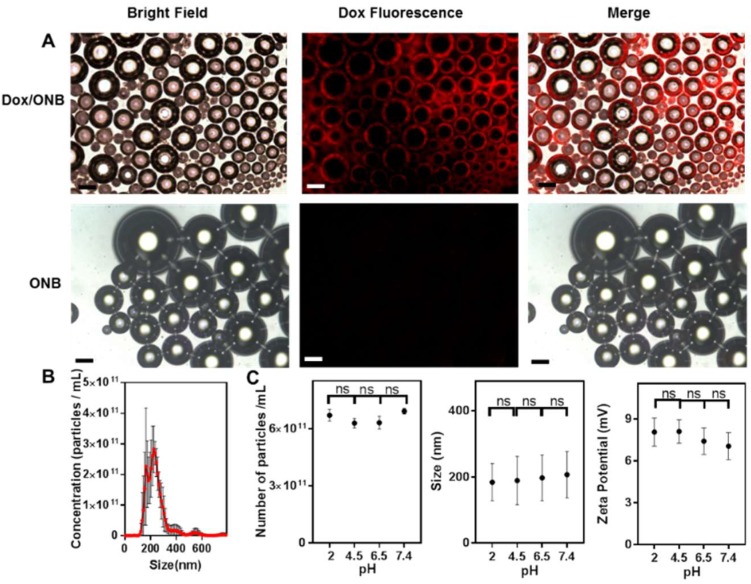
Characterization of Dox-encapsulated microsized and nanosized bubbles. (**A**) Bright field, fluorescence, and merged images of Dox-encapsulated microbubbles and ONB (without Dox) in a total solution containing both nanoscale and microscale bubbles. Scale bar = 20 µm. (**B**) Size distribution and concentration obtained by nanoparticle tracking analysis (NTA) for Dox-conjugated oxygen nanobubbles (Dox/ONB). (**C**) Comparison of the number of particles, size, and zeta potential of ONBs at different pH values. ns: no significant difference.

Figure 2 shows the characterization of Dox/ONBs. As shown in Figure 2A, microsized bubbles were collected before filtration, and Dox fluorescence was visualized using fluorescence microscopy to determine the shell/core composition of Dox/ONBs. The fluorescence images reveal the presence of Dox in the shell of the bubbles Comparatively, only ONBs show no fluorescence in the shell. Therefore, it is plausible to assume that Dox is encapsulated inside the shell of the ONBs. Although these bubbles are microsized, a similar phenomenon of Dox encapsulation is likely to happen in nanobubbles. Figure 2B shows the data obtained by NTA for particle size distribution and the number of particles/mL after filtering microscale bubbles. Most Dox/ONBs are in a size range of 150–300 nm, while the mean size is 258 ± 64 nm. The number of particles in Dox/ONB is (6.94 ± 0.73) × 10^11^ nanobubbles/mL. The Dox concentration in Dox/ONBs was measured by its fluorescence intensity and back-calculated as 130 µg/mL. Figure 2C shows a comparison of the number of particles, size, and zeta potential of ONBs at different pH values. These results indicate that there is no significant difference in the number of particles, size, and zeta potential of ONBs at different pH values. The number of particles are (6.2 ± 0.31) × 10^11^, (6.29 ± 0.25) × 10^11^, (6.31 ± 0.34) × 10^11^ and (6.91 ± 0.16) × 10^11^ nanobubbles/mL for pH values of 2, 4.5, 6.5, and 7.4, respectively. The particle size is (184 ± 56), (187 ± 73), (197 ± 69), and (207 ± 70) nm for the pH values of 2, 4.5, 6.5, and 7.4 respectively. The zeta potential has mean values of 8.04, 8.08, 7.39, and 7.03 mV at pH values of 2.5, 4.5, 6.5, and 7.4, respectively. These results indicate that the variation in the number of particles, size, and zeta potential across a pH gradient is insignificant for ONBs, and variation in the pH of the tumor microenvironment will not have a significant impact on the stability, size, zeta potential, and number of ONB particles.

**Figure 3:**
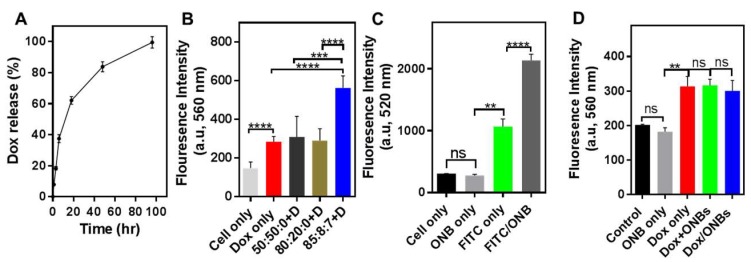
Drug release and cell penetration of the drug/ONB conjugates. (**A**) The drug release profile of Dox from Dox/ONB conjugates. (**B**) Cell penetration of the Dox + ONB mixture in MDA-MB-231 cells. Various lipid ratios (1,2-distearoyl-sn-glycero-3-phosphocholine (DSPC), 1,2-distearoyl-sn-glycero-3-phosphoethanolamine-N-[amino(polyethylene glycol)-2000] (DSPE-PEG-2000-Amine), and DSPE-PEG-2000-Biotin) were used to synthesize ONBs to assess their penetration effect, and were evaluated using the fluorescence intensity of Dox. (**C**) Cell penetration of FITC-conjugated ONBs in MDA-MB-231 cells evaluated using the fluorescence intensity of fluorescein (FITC) (**D**) Cell penetration of Dox/ONB was evaluated and compared to those of Dox only and Dox + ONB mixture in MDA-MB-231 cells. ** indicates *p*-value < 0.01, *** indicates *p*-value < 0.001, and **** indicates *p*-value < 0.0001. ns: no significant difference.

Figure 3 presents the results for the drug release and cell penetration. As shown in Figure 3A, 37.4% of the drug is released from Dox/ONBs in the first 6 h, and 62% of the drug is released after 18 h. These results indicate that the drug can be successfully released from Dox/ONB conjugates without the application of ultrasound because of the gas diffusion out of the ONB and release of Dox through diffusion and bubble collapse. Figure 3B shows the results of an experiment conducted to determine the optimal combinations of lipids for penetration into cancer cells. DSPC, DSPE-PEG-2000-Amine, and DSPE-PEG-2000-Biotin were each used to synthesize ONBs at ratios of 50:50:0, 80:20:0 and 85:8:7. These ONBs were then co-administered with the same amount of Dox by simply mixing Dox and ONBs. The best cellular penetration is obtained when all three lipids were used at an 85:8:7 molar ratio. Based on this result, we used this ratio for subsequent Dox/ONB conjugate preparations. Figure 3C shows the results of fluorescent ONB penetration into cancer cells. FITC-conjugated ONBs were used to visualize the effect of ONB penetration into cells. FITC/ONBs exhibit a two-fold higher fluorescence compared to that of FITC only, demonstrating that ONBs penetrated the cells. This experiment provides additional evidence of cellular uptake of ONBs. The fluorescence intensities of Dox/ONBs, Dox + ONB mixture, or free Dox after addition to the cellular media were compared. As shown in Figure 3D, there are no significant differences in fluorescence after 3 h of incubation. However, it is evident that Dox/ONB conjugates successfully deliver the drug to tumor cells in a similar manner to free Dox.

**Figure 4:**
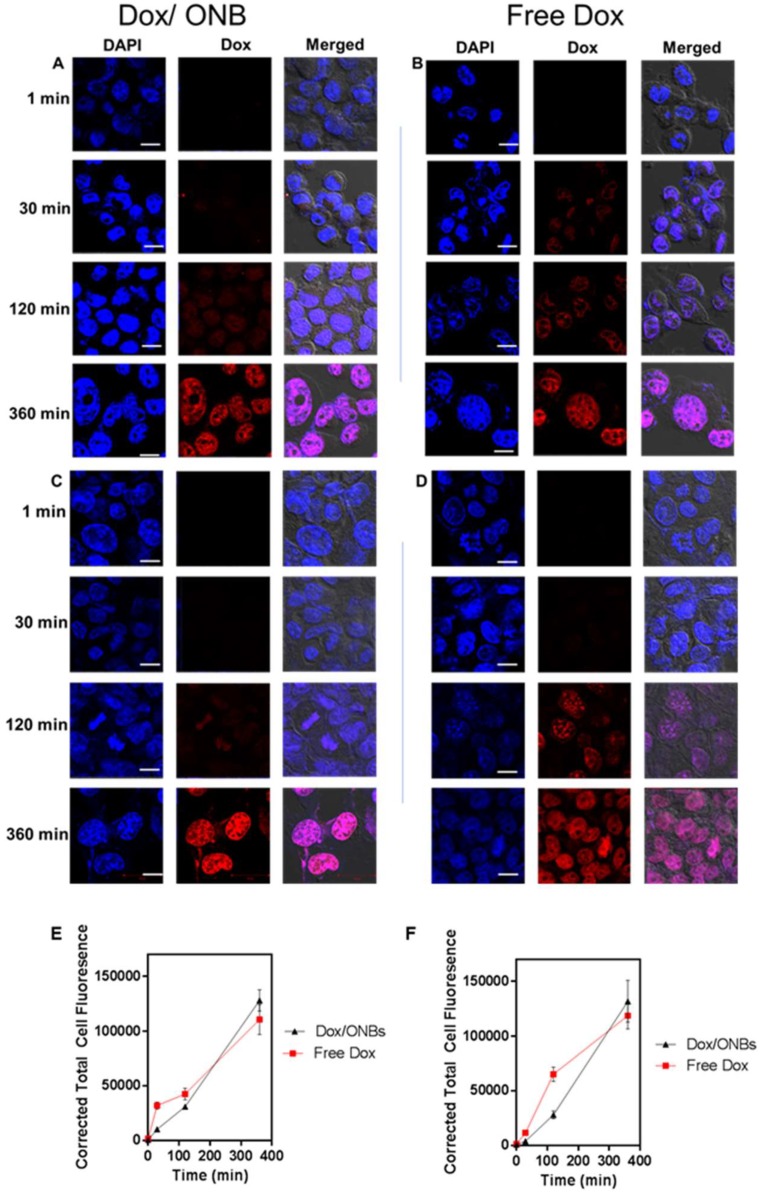
Intracellular confocal microscopy imaging of Dox penetration using Dox fluorescence and DAPI staining. Dox penetration in MDA-MB-231 cells using (**A**) Dox/ONB conjugates and (**B**) Free Dox. Scale bars = 10 µm. Dox penetration in HeLa cells using (**C**) Dox/ONB and (**D**) Free Dox. Scale bars = 10 µm. (**E**) Corrected total cell fluorescence intensity of Free Dox and Dox/ONBs for MDA-MB-231 cells from confocal images measured using ImageJ software. (**F**) Corrected total cell fluorescence intensity of free Dox and Dox/ONBs for HeLa cells from confocal images measured using ImageJ software.

Figure 4A,B presents intracellular confocal microscopy images of MDA-MB-231 cells, while Figure 4C,D represents intracellular confocal microscopy images of HeLa cells, clearly showing that Dox penetrated the cells rapidly and that fluorescence was visible after 30 minutes in both cell lines in the case of free Dox. Dox/ONB conjugates were slow to penetrate the cells, which is understandable because of the size of the molecules and endocytosis processes involved in uptake. Longer uptake times improved the biodistribution process. Despite the slow uptake of Dox/ONBs, after 6 h, the accumulation of Dox inside cells from Dox/ONBs improves. These results were quantified with Image J software and presented in Figure 4E,F for MDA-MB-231 cells and HeLa cells, respectively. Corrected total cell fluorescence from the confocal images indicated that the penetration of free Dox is significantly higher than that of Dox/ONBs in both MDA-MB-231 and HeLa cells. However, after 6 h, the Dox/ONB conjugates exhibit higher mean values, although the difference was not significant. Thus, despite a slow initial release of Dox from Dox/ONB, an almost equal amount of drug is delivered to the cell after 6 h, indicating successful Dox delivery from Dox/ONB.

**Figure 5:**
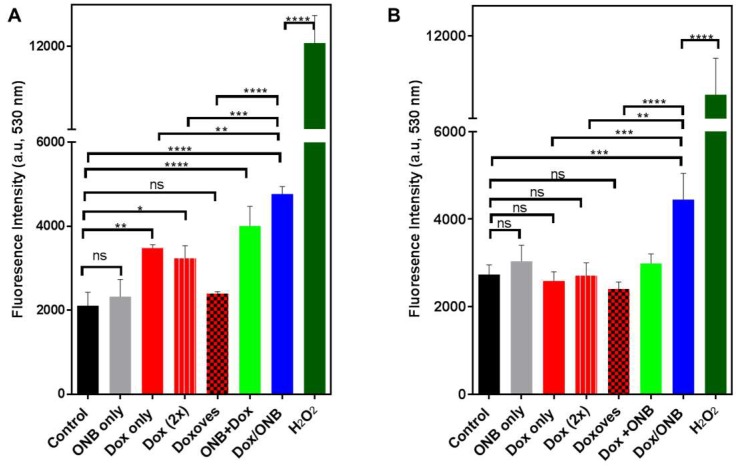
Dox uptake mechanism and efficacy test using the reactive oxygen species (ROS) assay kit. H_2_O_2_ was used as a positive control. (**A**) ROS assay fluorescence for MDA-MB-231 cells. (**B**) ROS assay fluorescence for HeLa cells. * indicates *p*-value < 0.05, ** indicates *p*-value < 0.01, and *** indicates *p*-value < 0.001. ns: no significant difference. Statistical analysis was done by using one-way ANOVA followed by post hoc analysis with Tukey’s multiple comparisons test.

Figure 5A,B demonstrate ROS generation in MDA-MB-231 and HeLa cells under various treatment conditions. The results for both cell types indicate that only ONBs without drug did not generate significantly higher ROS levels compared with that in the non-treated control samples.

In MDA-MB-231 cells, Dox and Dox (2×) generate a higher level of ROS compared to control, but those levels are significantly lower compared to Dox/ONB. The mean fluorescence intensity of Dox/ONB is 4767, which is almost twice as high that of Doxoves, which has a mean fluorescence intensity of 2399, indicating the effect of the combined presence of oxygen and doxorubicin on ROS generation. In HeLa cells, ROS generation in response to ONB, Dox, Dox (2×), or Doxoves is not significantly higher than control, while in response to Dox/ONB, a significantly higher level of ROS (mean value 4446.5) was generated. The values for the other treatments are: Doxoves (mean value 2411), Dox (2×) (mean value 2711), and Dox (mean value 2588). H_2_O_2_ was used as a positive control. Taken together, these results demonstrate that the presence of ONBs is not harmful, and that ROS are not generated if the tumor-derived cells are exposed to only ONBs. In addition, increasing the amount of Dox (as in cells exposed to Dox (2×)) or exposing the cells to commercially-available liposomal Dox (Doxoves) generated lower ROS than Dox/ONB. Therefore, it is evident that higher levels of ROS are generated in the combined presence of Dox and oxygen in the case of Dox/ONB, which could improve the efficacy of the drug.

**Figure 6:**
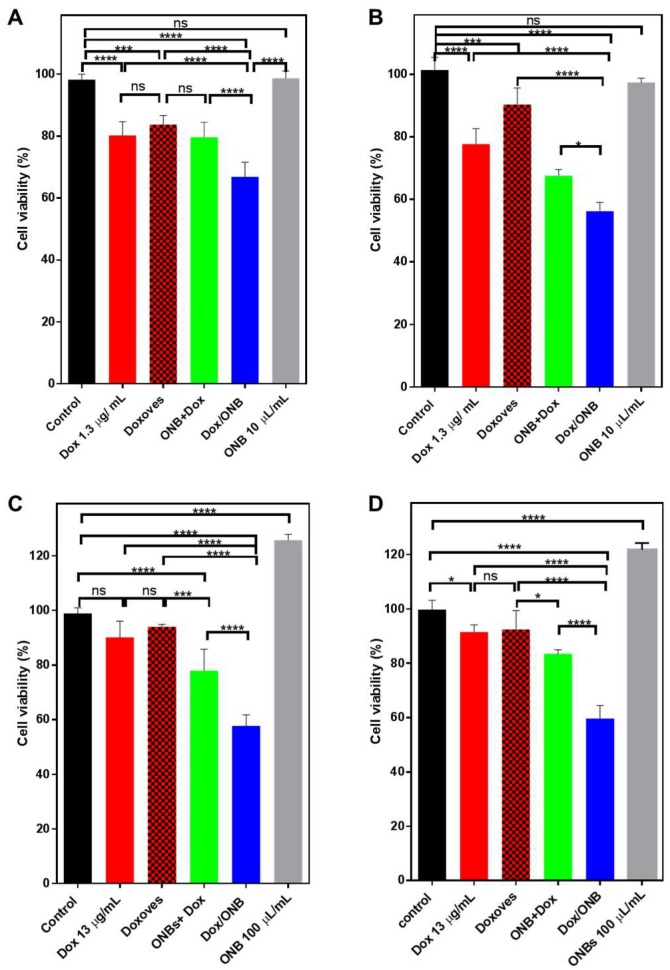
Cell viability and Dox efficiency measured in normal conditions and hypoxic conditions. Cell viability measured through trypan blue staining, control normalized at 100%. (**A**) Normal condition MDA-MB-231 cell line. (**B**) Normal conditions HeLa cell line (**C**) Cell viability in hypoxic conditions for MDA-MB-231 cells (**D**) Cell viability in hypoxic conditions for HeLa cells. * indicates *p*-value < 0.05, *** indicates *p*-value < 0.001, **** indicates *p*-value < 0.0001 ns indicates no significant difference. Statistical analysis was done by using one-way ANOVA followed by post hoc analysis of Tukey’s multiple comparisons test.

The results shown in Figure 6 demonstrate the effectiveness of Dox/ONBs under normal incubation conditions and hypoxic conditions. MDA-MB-231 cells and HeLa cells were grown to maximum confluence for these experiments. Since 10 µL of Dox/ONB contains 1.3 µg of Dox, the same amount of Dox was used to compare between free Dox, Doxoves, the ONB + Dox mixture, and Dox/ONB.

In MDA-MB-231 cells (Figure 6A), the cell viability with Dox was 80.25%; with Doxoves, it was 83.68%; and with Dox + ONB, it was 79.6%. These values are significantly lower than the non-treated controls, but among them, there is no significant difference. Cell viability with Dox/ONB conjugates was 66.8%, which is significantly lower than the other values. This trend can be explained by our previous results for ROS generation, demonstrating that Dox/ONB conjugates are more effective drug carriers. In Figure 6B, the in vitro results with HeLa cells in normal conditions are presented. Free Dox reduces the cell viability to 77%, while Doxoves shows a cell viability of 90%. The Dox + ONB mixture shows a cell viability of 67.5%, while the use of Dox/ONB conjugates results in significantly lower cell viability: 56%. Only ONBs show cell viability that is similar to non-treated controls. These results under normal conditions show that Dox/ONB is able to reduce cell viability and prove effective in treatment, owing to cellular uptake and ROS generation.

Under hypoxic conditions, ONBs and Dox/ONBs were used at higher concentrations (10% of media volume) compared to those used under normal conditions. Therefore, the amount of free Dox and Doxoves was also increased to match the concentration. First, the cell lines were exposed to hypoxic conditions for 6 h; after treatment, the cells were kept under normal conditions. Pretreatment exposure to hypoxia was aimed at the expression and stabilization of the HIF-1α protein. The results of treatment under hypoxic conditions are shown in Figure 6C,D. As shown in Figure 6C, the cell viability of the control sample was normalized to 100%. ONBs increase the cell viability to 125%, which is significantly higher than the non-treated control. Free Dox and Doxoves, even when used at a higher concentration than that used under normal conditions, do not significantly increase cytotoxicity compared to that in the negative control. Dox + ONB shows a cell viability of 77.9%, which is significantly lower than that of Doxoves (93.9%). Dox/ONB conjugates are effective under hypoxic conditions, reducing cell viability to 57.7% in MDA-MB-231 cells, which is a significant decrease compared with all other categories.

Similar results are seen in Figure 6D for HeLa cells, where Dox/ONBs reduce the cell viability to 60% compared to 91% with free Dox and 92.4% with Doxoves. In both cases, Dox/ONBs successfully reduce cell viability in 24 h. This result establishes that Dox/ONBs are effective under hypoxic conditions and reduce the impact of HIF-1α, which is an impact that is seen with free Dox and Doxoves.

## 4. Discussion

In our previous study related to ONBs, we discussed the surface composition of ONBs, as well as their biocompatibility, stability, ability to deliver oxygen to hypoxic areas, and ability to downregulate HIF-1α protein [32,38]. In this study, we demonstrated that Dox/ONBs effectively delivered Dox and oxygen into MDA-MB-231 breast cancer and HeLa cervical cancer cells. Dox was successfully encapsulated in ONBs with a particle size range of approximately 150–300 nm with a mean size of 258 ± 64 nm. This mean size is in the optimal range to utilize the EPR effect and achieve better penetration into solid tumors. ONBs exhibited no significant changes when they were dissolved in buffers having pH values ranging from 2 to 7.4, and the size, number of particles, and zeta potential also remained similar across this pH gradient. Since the tumor microenvironment is associated with acidic pH values, the stability of ONBs at different pH values is critical. Dox is a lipophilic compound and has an affinity for phospholipids; therefore, liposomal Dox is commercially available. We used Doxeves (liposomal Dox) as a reference to evaluate the effect of the oxygen component of Dox/ONB. Fluorescence microscopy of the inherent Dox fluorescence revealed the shell-core composition and the presence of Dox in the shells of microsized bubbles. Although some research groups have used centrifugation to remove unconjugated Dox, we opted for dialysis to remove unbound Dox. After dialysis of Dox/ONBs against deionized water for two days, the Dox concentration conjugated with ONBs was 130 µg/mL, as calculated from a Dox standard curve. Thus, it was estimated that each nanobubble contained 0.18 ng of Dox. The loading capacity of Dox/ONBs can be increased by altering the shell composition. The drug release profile indicated that almost all of the drug was released from ONBs in 4 days without using ultrasound. This complete drug release can be attributed to the diffusion process and rupturing of nanobubbles due to external Laplace pressure after oxygen leaves the core. Therefore, Dox/ONB can be used for drug delivery without ultrasound.

Cell penetration tests showed that Dox/ONBs successfully penetrated the cells in amounts similar to or higher than free Dox. FITC-conjugated ONBs had a higher fluorescence intensity, confirming the successful delivery of ONBs to the cells. Confocal microscopy imaging of MDA-MB-231 and HeLa cells revealed that free Dox penetrated significantly faster than Dox/ONBs, but after 6 h, relatively brighter images of Dox fluorescence with Dox/ONB conjugates were observed. The release mechanism, as discussed earlier, can be attributed to the diffusion and rupturing of ONBs. After cellular uptake, lipid shells are metabolized, releasing Dox and oxygen. Some nanobubbles might release Dox outside the cell, but eventually, it would penetrate the cell. Confocal imaging of DAPI staining showed that Dox fluorescence of Dox/ONBs was concentrated in the nucleus, indicating the successful Dox delivery. The longer cellular uptake duration for Dox/ONBs is related to the better biodistribution and bioavailability of Dox.

To evaluate the ROS generation of Dox/ONB, we performed ROS assays and compared the ROS generation capability of Dox/ONB with free Dox, Dox (2×), Doxoves, and Dox + ONB. The results showed that Dox/ONBs generated significantly higher ROS, while ONBs failed to increase ROS generation. Free Dox and a double dosage of free Dox, which is represented as Dox (2×), generated significantly lower ROS than Dox/ONB. This shows that, even at double dose, the ROS generation of free Dox is low compared with that of Dox/ONB. ROS generation in response to Doxoves was almost half that of Dox/ONB. Therefore, we conclude that treatment with ONBs alone is not threatening to the cells, but when Dox is encapsulated in ONBs, the synergetic effect of Dox and oxygen results in higher ROS generation. The ROS mechanism of Dox/ONB is an important factor in enhancing the therapeutic action of the drug.

The cell viability experiment was aimed at evaluating the effectiveness of Dox/ONB in normal and hypoxic conditions. Under normal incubation conditions, Dox/ONB was more effective in reducing cell viability in 24 h compared with Dox and Doxoves in MDA-MB-231 and HeLa cancer cell lines, while ONB was not cytotoxic for cells.

We previously reported that the exposure of tumor cells to hypoxic conditions upregulates and stabilizes HIF-1α, while ONBs are effective in reversing hypoxia and downregulating HIF-1α [38]. The overexpression and stabilization of HIF-1α have been associated with resistance to Dox by many researchers [13,14,15,16,17,20,83,84,85,86]. In the current study, cells treated under hypoxic conditions were more resistant to free Dox, but were effectively killed by Dox/ONB conjugates. The difference in cell viability between the non-treated control, free Dox, and Doxoves groups was not significant in MDA-MB-231 or Hela cells, while Dox/ONB conjugates significantly decreased cell viability to 57.7% in MDA-MB-231 cells and 60% in HeLa cells. This lower cell viability indicates that Dox/ONB conjugates have the potential to more effectively treat tumors under hypoxic conditions, and the higher efficacy of Dox/ONBs can also be attributed to the downregulation of HIF-1α. ONBs were able to increase cell viability in both cell lines, which is an indication that cellular conditions improved and the degradation of HIF-1α protein occurred. The Dox + ONB mixture was added to these experiments to evaluate the effect of the encapsulation of Dox in Dox/ONB. The Dox + ONB mixture refers to the co-administration of Dox and ONB. Our results under normal and hypoxic conditions showed that Dox/ONB outperformed the Dox + ONB mixture, showing evidence of the successful encapsulation of Dox into ONB.

Three-dimensional (3D) cell cultures are extensively used for mimicking tumor microenvironments, and researchers have shown that in 3D spheroid culture systems, the core of the tumor spheroid becomes hypoxic when it grows beyond a specific size limit (>500 µm) [87,88]. Dox/ONBs might improve tumor treatment in 3D models. Treatment modalities such as photodynamic therapy and radiation therapy are also highly dependent on the presence of oxygen at the tumor site. Even a temporary increase in oxygen levels may cause an increase the radiation sensitivity of tumors, whereas photodynamic therapy, which is dependent on the generation of reactive oxygen species (ROS), also benefits from increased oxygen levels at tumor sites [40,89].

In summary, Dox/ONB proved to be more effective under normoxic and hypoxic conditions than free Dox, Doxoves, and a Dox + ONB mixture. These results indicate the successful bioconjugation of Dox/ONBs and the effective delivery of Dox and oxygen to the tumor-derived cells in culture. We anticipate that ONBs can carry different therapeutic molecules on their surface in sufficient amounts to target tumor cells. Also, we anticipate that the size of ONBs size can be controlled by adjusting the combinations of lipid mixtures comprising ONBs, which in turn modulates the number of therapeutic molecules that can be loaded and the efficacy of cell penetration. These modalities would facilitate the application of ONBs to deliver oxygen and therapeutic molecules into 3D spheroids or tissues. For human applications, Dox/ONBs could be directly injected into the tumor or delivered intravenously. The ERP effect can be utilized by Dox/ONB to accumulate in tumors. Our results indicated a higher treatment efficacy than commercially available Doxoves; therefore, it is plausible that Dox/ONB would be more effective in tumor treatment.

## 5. Conclusions

Oxygen nanobubbles have previously been reported to reverse of hypoxia and downregulate HIF-1α. In this study, we proposed a novel idea to load Dox into oxygen nanobubbles to downregulate HIF-1α, enhance ROS generation, and reduce the amount of Dox required to achieve the intracellular effects that are associated with its therapeutic action. We compared Dox/ONBs with commercially available liposomal Dox (Doxoves) and free Dox, and found out that Dox/ONBs were more effective in tumor cells cultured under normal and hypoxic conditions (i.e., breast cancer MDA-MB-231 and cervical cancer HeLa cell lines). ROS assays and cytotoxicity assays showed the effectiveness of Dox/ONB. Further investigations are required to establish the in vivo and clinical effects of this proposed model.
